# Urinary Tract Infectious Complications After Retrograde Intrarenal Surgery: The RIRS-STAMP Risk Score from a Two-Center Cohort

**DOI:** 10.3390/pathogens15050471

**Published:** 2026-04-27

**Authors:** Mehmet Ozturk, Huseyin Cihan Demirel, Ilker Seckiner, Taner Haciosmanoglu, Muharrem Baturu, Semih Turk, Onur Zeytun, Kaya Horasanli

**Affiliations:** 1 Department of Urology, Gaziantep University, Gaziantep 27310, Türkiye; 2 Department of Urology, İstanbul Aydın University, İstanbul 34295, Türkiye; 3 Department of Urology, Sehitkamil Public Hospital, Gaziantep 27060, Türkiye; 4 Department of Urology, Murat Public Hospital, Edirne Sultan 1, Edirne 22030, Türkiye; 5 Department of Urology, İstanbul Şişli Hamidiye Etfal Research Center, University of Health Sciences, İstanbul 34381, Türkiye

**Keywords:** retrograde intrarenal surgery, urinary tract infection, urosepsis

## Abstract

Introduction: Postoperative infectious complications following retrograde intrarenal surgery (RIRS) remain a significant clinical challenge due to their potential progression to sepsis. This study aimed to identify perioperative risk factors associated with infection and to develop a practical risk stratification model. Materials and Methods: A total of 1949 patients who underwent RIRS in two centers between 2014 and 2025 were retrospectively analyzed. Patients were grouped according to irrigation method, and infectious outcomes including febrile urinary tract infection (UTI), sepsis, and septic shock were evaluated. Multivariable logistic regression analysis identified independent predictors of postoperative infection. Results: Overall, infectious complications occurred in 158 patients (8.1%), with no significant difference in total infection rates between the two groups. In the overall cohort, older age (OR 1.01; *p* = 0.045), preoperative JJ stenting (OR 1.48; *p* = 0.038), longer operative time (OR 1.01; *p* = 0.049), and a history of preoperative UTI requiring antibacterial treatment (OR 68.45; *p* < 0.001) were independent predictors of postoperative infection. These variables informed the development of the RIRS-STAMP score; the final combined model showed good discrimination (AUC 0.84, 95% CI 0.80–0.88) and was internally validated using 1000 bootstrap resamples. Discussion: These findings highlight the multifactorial nature of infection risk after RIRS and emphasize the importance of both host factors and procedural dynamics in infection development. The RIRS-STAMP score was developed based on these findings. Conclusions: The model can enable early identification of high-risk patients and supports individualized perioperative management; however, prospective external validation is required before routine clinical use.

## 1. Introduction

Retrograde intrarenal surgery (RIRS) has emerged as a preferred minimally invasive approach for renal calculi management due to its low morbidity, high stone-free rates, rapid recovery, and short hospital stay [1,2]. However, postoperative infectious complications—including febrile urinary tract infection (2.8–17.5%) and urosepsis (0.5–11.1%)—remain clinically significant, with overall complication rates ranging from 5% to 25% [3,4,5]. These complications are thought to be closely related to increased intrarenal pressure during RIRS, which promotes pyelovenous and pyelotubular reflux, facilitating bacterial translocation into the systemic circulation [6].

Identifying factors associated with postoperative infection is essential for improving risk stratification and guiding preventive strategies. Previous studies have highlighted several clinical risk factors, including diabetes, hydronephrosis, prior urinary tract infections, positive preoperative urine cultures, increased stone burden, multiple stones, and prolonged operative time [7,8]. Nevertheless, the pathogenesis of infection after RIRS is complex and likely reflects an interplay between host susceptibility, procedural dynamics, and microbial factors.

Preoperative urine culture and targeted antibiotic prophylaxis remain cornerstones of infection prevention. However, bacterial colonization within stones and biofilm formation on indwelling devices, such as ureteral stents, may lead to false-negative culture results and persistent microbial reservoirs [8,9]. These findings underscore the importance of considering both host–pathogen interactions and occult microbial burden when evaluating infection risk. In this context, strategies such as minimizing operative time, optimizing irrigation pressure, routine use of ureteral access sheaths (UAS), a staged approach in selected high-risk patients, such as those with a large stone burden or difficult access, to avoid prolonged operative time and the associated increased risk of infection after RIRS [10,11].

During RIRS, the irrigation technique may influence intrarenal pressure and, consequently, the risk of infection [11,12]. In the present study, because irrigation modalities completely overlapped with the study centers (i.e., there was complete confounding), the independent effect of the irrigation technique could not be reliably compared, and the observed differences could not be attributed solely to the irrigation method. Therefore, findings related to irrigation should be interpreted as exploratory and hypothesis-generating rather than causal.

Therefore, the aim of this study was to identify preoperative and intraoperative clinical variables associated with postoperative infectious complications following RIRS and to develop a clinically applicable risk stratification model to support individualized infection prevention strategies.

## 2. Materials and Methods

### 2.1. Study Design and Population

We retrospectively analyzed the medical records of 1949 adult patients who underwent RIRS at two different tertiary centers from February 2014 to January 2025. The inclusion criteria were: (1) age ≥ 18 years; (2) undergoing RIRS during the study period at either center; and (3) availability of complete demographic, perioperative, and postoperative data in the institutional electronic medical records. Patients with horseshoe kidney, ureteropelvic junction obstruction, neurogenic bladder, active systemic infection, renal transplantation, or those undergoing combined procedures, including endoscopic combined intrarenal surgery (ECIRS), were excluded. After application of the eligibility criteria, including 1112 patients in Group 1 and 837 patients in Group 2.

**Figure 1:**
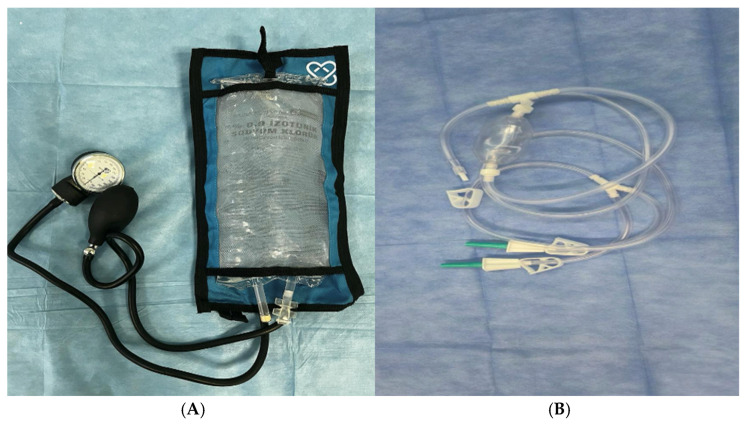
(**A**): Pressure irrigation pump. (**B**): Hand pump.

Ethical approval was obtained from the institutional review board (decision no. 396) and data collection commenced only after approval had been granted. Patients were classified according to the intraoperative irrigation method used during RIRS. Group 1 underwent RIRS using a gravity-driven irrigation system with pressure bags fixed at 100 cmH_2_O, whereas Group 2 underwent RIRS using a manual irrigation pump delivering 1–10 mL of fluid per compression (Figure 1). Because each irrigation modality was used exclusively at a different tertiary center, study center and irrigation method were inseparable in the present cohort. Therefore, comparisons between the two groups were interpreted as descriptive rather than causal, and any observed differences in postoperative infectious outcomes cannot be attributed solely to the irrigation technique, as center-related factors may also have contributed. Demographic characteristics, perioperative variables, and stone-related parameters were analyzed to identify predictors of postoperative infectious complications. All available preoperative urine culture results were reviewed retrospectively. For culture-positive specimens, isolated microorganisms were recorded and reported descriptively by study group. Polymicrobial cultures were documented separately. Species-specific statistical comparisons were not performed because the microbiological data were isolate-based and several categories contained only a small number of isolates.

### 2.2. Definition of Stone-Free Status

Stone-free status was defined as either complete absence of residual fragments or the presence of clinically insignificant residual fragments (CIRF). CIRF were defined as fragments ≤4 mm in maximum diameter on non-contrast computed tomography (NCCT). In patients with multiple residual fragments, cumulative stone burden was calculated as the sum of the maximum diameters of all fragments.

### 2.3. Definition of Urinary Tract Infectious Outcomes

The primary outcome of the study was postoperative urosepsis following RIRS, with septic shock defined separately as its most severe manifestation. Urosepsis was defined as sepsis of urinary tract origin according to the Sepsis-3 criteria. Because of the retrospective design, cases were identified based on a physician-documented diagnosis supported by objective indicators of infection-related organ dysfunction, including vasopressor use, elevated serum lactate levels, and/or intensive care unit admission. Septic shock was defined as persistent hypotension requiring vasopressor therapy despite adequate fluid resuscitation. Secondary infectious outcomes were early postoperative febrile urinary tract infection (UTI), defined as a body temperature ≥ 38 °C within 48 h after surgery with supporting clinical and/or laboratory findings, and late febrile UTI, defined as infection occurring >48 h after surgery resulting in prolonged hospitalization or hospital readmission.

### 2.4. Surgical Procedure

Before stone treatment, urine culture and urinary microscopy findings were evaluated; when a urinary tract infection was detected, culture-directed therapy was administered before intervention. Perioperative antibiotic prophylaxis for RIRS consisted of ceftriaxone 1 g as a single dose in Group 1 and cefuroxime axetil 1.5 g in Group 2, selected according to the institutional antibiogram and local antimicrobial susceptibility profile. The variable ‘recent preoperative UTI requiring therapeutic antibiotics’ referred to a documented symptomatic UTI treated before surgery and did not denote routine perioperative prophylaxis. RIRS was performed under general anesthesia in the lithotomy position by experienced surgeons (>100 cases).

Pre-existing JJ stents were removed via cystoscopy, followed by ureteral access using rigid ureterorenoscopy and placement of an 11/13-Fr ureteral access sheath (Navigator™, Boston Scientific, Marlborough, MA, USA). In cases where sheath placement was not feasible, flexible ureterorenoscopy was performed over a guidewire.

Stone fragmentation was performed using either holmium: YAG laser (0.6–1.5 J, 8–15 Hz) or thulium fiber laser (0.05–0.15 J, 100–300 Hz) for dusting and fragmentation. Total operative time was defined as the duration from cystoscopy initiation to bladder catheterization.

### 2.5. Statistical Analysis

Statistical analyses were conducted primarily using SPSS version 29.0 (IBM Corp., Armonk, NY, USA). Continuous variables were summarized as mean ± standard deviation or median (interquartile range), according to data distribution, and categorical variables were presented as frequencies and percentages. Between-group comparisons were performed using the chi-square test or Fisher’s exact test for categorical variables and the independent-samples *t* test or Mann–Whitney U test for continuous variables, as appropriate. The predefined postoperative infectious outcome was used as the dependent variable for model development. Model derivation and internal validation were performed using complete-case analysis. Independent predictors of postoperative infectious complications were identified using multivariable logistic regression with clinically preselected covariates, and results were expressed as odds ratios (ORs) with 95% confidence intervals (CIs). Multicollinearity was assessed using the variance inflation factor (VIF), and overall model fit was described using Nagelkerke’s R^2^. A point-based risk score (RIRS-STAMP) was derived from the final multivariable model by assigning integer points proportional to the regression coefficients. Model discrimination was evaluated using the area under the receiver operating characteristic curve (AUC) with 95% CIs, and calibration was assessed using a calibration plot and the Hosmer–Lemeshow goodness-of-fit test. Internal validation was performed using 1000 bootstrap resamples to estimate optimism-corrected model performance. For clinically relevant score thresholds or predefined risk tiers, class-specific performance metrics, including sensitivity, specificity, positive predictive value, and negative predictive value, were calculated with 95% CIs. Clinical utility was further assessed using decision curve analysis by comparing the net benefit of the model with treat-all and treat-none strategies across a range of threshold probabilities. A two-sided *p* value < 0.05 was considered statistically significant.

## 3. Results

A total of 1949 patients were included in the analysis, including 1112 (57.1%) treated with a gravity-driven pressure bag (Group 1) and 837 (42.9%) treated with a manual irrigation pump (Group 2). Compared with Group 2, Group 1 more frequently underwent preoperative JJ stenting (95% CI, 29.4–37.5; *p* < 0.001), received antibiotic treatment for preoperative urinary tract infection (95% CI, 5.5–9.9; *p* < 0.001), and achieved a higher stone-free rate (95% CI, 2.5–8.1; *p* < 0.001). By contrast, patients in Group 2 were older (95% CI, 4.17–7.12; *p* < 0.001) and had a slightly larger median stone size (17 [IQR, 12–23] vs. 15 [IQR, 10–24] mm). Operative duration was shorter in Group 1 (median 40 [IQR, 25–60] vs. 60 [IQR, 45–70] min), which was reflected by a higher proportion of procedures completed within 60 min (95% CI, 20.9–29.5; *p* < 0.001). Sex distribution and stone location also differed between groups, whereas laterality, access sheath use, postoperative JJ stenting, length of hospital stay, preoperative urine culture positivity, and infectious complications were similar (Table 1).

Available preoperative culture data showed 49 isolates from 47 culture-positive samples in Group 1 and 35 isolates from 27 culture-positive samples in Group 2. In both groups, *Escherichia coli* was the most frequently isolated microorganism (Group 1: 21/49, 42.9%; Group 2: 12/35, 34.3%). In Group 1, *E. coli* was followed by *Klebsiella pneumoniae* (9/49, 18.4%) and *Pseudomonas aeruginosa* (5/49, 10.2%), whereas in Group 2 the next most common organisms were *Pseudomonas aeruginosa* (7/35, 20.0%) and *Enterococcus faecalis* (6/35, 17.1%). Polymicrobial cultures were significantly more frequent in Group 2 than in Group 1 (7/27, 25.9% vs. 2/47, 4.3%; *p* = 0.010) (Table A1).

Overall, infectious complications occurred in 158 patients (8.1%), with no significant difference between the two groups (8.54% vs. 7.53%, *p* = 0.451). However, the pattern of infection differed between groups. Late-onset infections were significantly more frequent in Group 1 (OR 1.65; *p* = 0.036), whereas major infectious complications, including sepsis and septic shock, were significantly lower in this group (OR 0.42; *p* = 0.016) (Table 2).

In the pooled analysis of Groups 1 and 2, infection-positive patients were significantly older, had larger stones, were more likely to have a preoperative JJ stent, and had longer operative times and hospital stay than infection-negative patients. Antibacterial treatment for preoperative UTI was also significantly more common in infection-positive patients (all *p* < 0.05; Table A2). In subgroup analyses, sex distribution differed significantly in both groups, with a female predominance among infected cases in Group 1 and a male predominance in Group 2. In Group 1, infection-positive patients were additionally older, had larger stones, were more likely to have stones ≥20 mm, had longer operative times and a higher proportion of procedures lasting ≥60 min, had longer hospital stay, and more frequently received antibacterial treatment for preoperative UTI (all *p* < 0.05). In Group 2, apart from sex distribution, only hospital stay and antibacterial treatment for preoperative UTI remained significantly different between infection-positive and infection-negative patients (all *p* < 0.05; Table A2).

Multivariable logistic regression analysis revealed distinct predictors across groups. In Group 1, female sex (OR 1.85; *p* = 0.016), larger stone size (OR 1.03 per mm; *p* = 0.008), and prior antibacterial treatment for preoperative UTI (OR 82.40; *p* < 0.001) were independent predictors of infection. In Group 2, male sex (OR 2.12; *p* = 0.010) and preoperative UTI treatment (OR 10.85; *p* < 0.001) were identified as independent predictors. In the overall cohort, independent predictors of postoperative infection included age (OR 1.01 per year; *p* = 0.045), presence of a preoperative JJ stent (OR 1.48; *p* = 0.038), operative time (OR 1.01 per minute; *p* = 0.049), and preoperative antibacterial treatment for UTI (OR 68.45; *p* < 0.001) (Table 3). No relevant multicollinearity was detected among the predictors included in the final multivariable model; VIF values ranged from 1.02 to 1.60 (age, 1.03; stone size, 1.57; preoperative JJ stent, 1.02; operative time, 1.60; and antibacterial treatment for preoperative UTI, 1.02).

**Figure 2:**
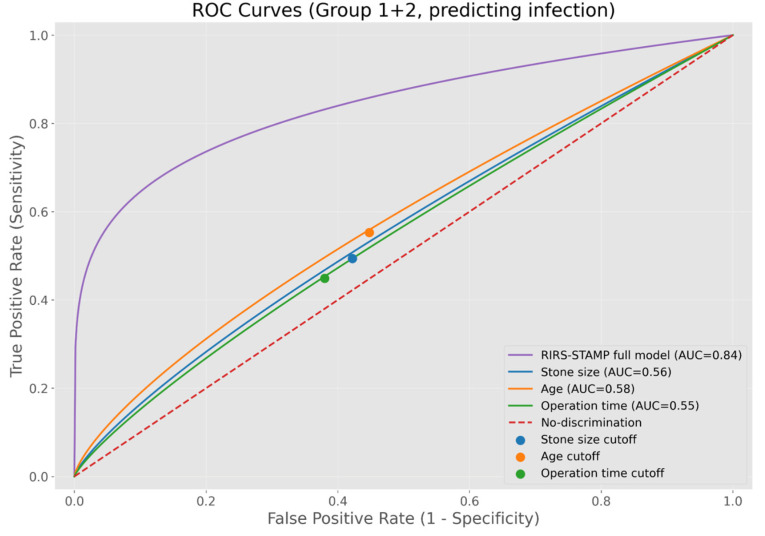
ROC curves of variables including age, stone size, and operation time in predicting the development of infection.

Receiver operating characteristic (ROC) analysis demonstrated that stone size (>19.5 mm), age (>43.5 years), and operative time (>59.5 min) were statistically significant predictors of infection, although with modest discriminative performance (AUC = 0.56, *p* = 0.013; AUC = 0.58, *p* = 0.003; AUC = 0.55, *p* = 0.041, respectively) (Figure 2, Table 4).

**Figure 3:**
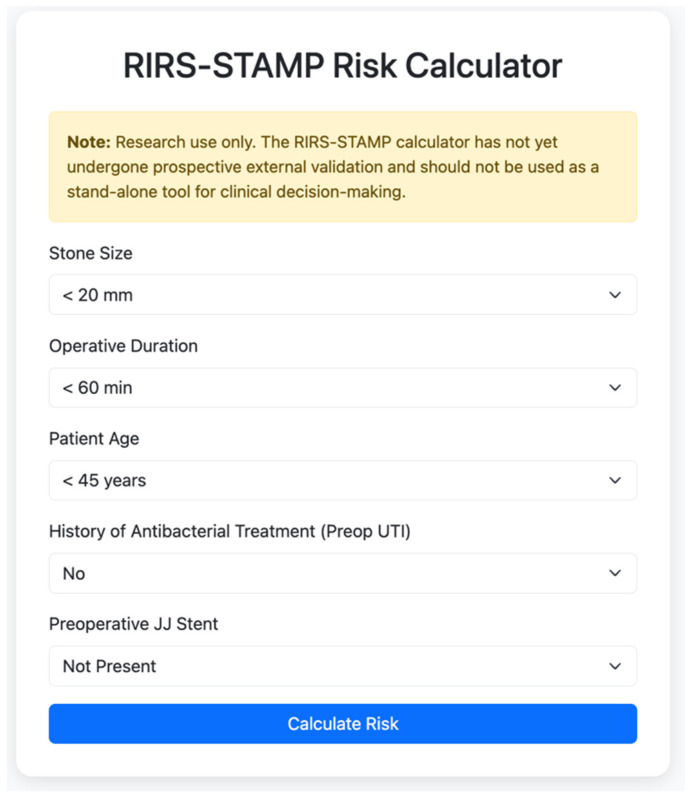
Web interface of the RIRS-STAMP scoring system (online calculator).

In the pooled cohort, multivariable logistic regression identified age, operative time, preoperative JJ stent placement, and antibacterial treatment for preoperative UTI as independent predictors of postoperative infectious complications. Although stone size lost statistical significance after multivariable adjustment, it was retained in the point-based model because of its clinical relevance and its univariable association with infection risk. Based on these findings, a simplified bedside risk score, the RIRS-STAMP score, was developed using clinically rounded thresholds for continuous predictors (Table 5). These findings informed the development of the RIRS-STAMP score, with clinical relevance; the final model showed good discriminative performance (AUC 0.84, 95% CI 0.80–0.88). Internal validation using 1000 bootstrap resamples demonstrated good discrimination and acceptable calibration of the final score. Calibration was acceptable (Hosmer–Lemeshow χ^2^ = 10.28, *p* = 0.246), and the calibration plot showed good agreement between predicted and observed risk. In addition, class-specific performance metrics showed that the higher score strata were associated with improved rule-in performance, particularly in the very high-risk category. For clinical applicability, the total score was grouped into four predefined risk strata, as shown in Table 5. For improved clinical applicability, a web-based calculator was developed and is publicly accessible (https://rirsstamp.pythonanywhere.com/) (accessed on 21 April 2026) (Figure 3).

**Figure A1:**
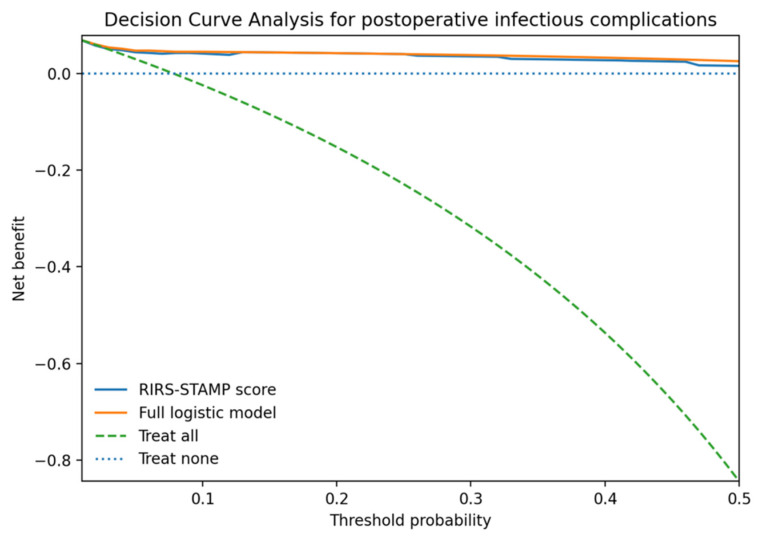
Decision curve analysis of the RIRS-STAMP score for predicting postoperative infectious complications in the pooled cohort.

Class-specific performance metrics showed that the very high-risk category had the strongest rule-in profile, with a specificity of 97.6%, a PPV of 70.5%, and an NPV of 96.8%. The high-risk category showed the most balanced sensitivity and specificity (68.4% and 72.8%, respectively). In contrast, the low-risk category was characterized by a high NPV (97.2%) but a low PPV (2.8%), supporting its potential use in identifying patients at relatively low risk of postoperative infectious complications (Table A3, Figure A1). According to the RIRS-STAMP score, 1033 patients (53.0%) were classified as low risk, 770 (39.5%) as moderate risk, 75 (3.9%) as high risk, and 71 (3.6%) as very high risk. The observed infection rates for each risk category are detailed in Table 5.

Observed and estimated postoperative infectious risk according to RIRS-STAMP score category (Table 6 and Table 7):

Note: Total RIRS-STAMP score was calculated by summing the points assigned to each variable. Point derivation was performed using a regression-calibrated integer-weight optimization approach. First, continuous predictors were dichotomized using ROC-informed and clinically rounded cutoffs: stone size ≥ 20 mm, operative time ≥ 60 min, and age ≥ 45 years. For each patient, the following binary indicators were defined: $S_{i}=1$ if stone size was ≥20 mm and 0 otherwise; $T_{i}=1$ if operative time was ≥ 60 min and 0 otherwise; $A_{i}=1$ if age was ≥45 years and 0 otherwise; $M_{i}=1$ if recent preoperative UTI requiring antibacterial treatment was present and 0 otherwise; and $J_{i}=1$ if a preoperative JJ stent was present and 0 otherwise.

For each candidate integer-weight vector $w=(w_{S},w_{T},w_{A},w_{M},w_{J})$, an additive candidate score was calculated as:

$$R_{i}(w)=w_{S}S_{i}+w_{T}T_{i}+w_{A}A_{i}+w_{M}M_{i}+w_{J}J_{i}$$

Because the logistic calibration model is scale-dependent for point presentation, recent preoperative UTI requiring antibacterial treatment, which was the strongest predictor in the multivariable model, was used as the scale anchor and assigned the maximum weight of 50 points. The remaining candidate integer weights were then evaluated using a logistic calibration model:

$$logit(p_{i})=\alpha_{w}+\theta_{w}R_{i}(w)$$

where $p_{i}$ represents the predicted probability of postoperative infectious complication. The optimal integer-weight vector was selected by minimizing the negative log-likelihood of the calibration model while preserving clinically interpretable and monotonically increasing risk strata:

$$w^{*}=arg\;\underset{w\in W}{min}[-\frac{1}{n}\sum_{i=1}^{n}\left\{y_{i}log({\hat{p}}_{i})+(1-y_{i})log(1-{\hat{p}}_{i})\right\}]$$

where $y_{i}=1$ indicates the occurrence of postoperative infectious complication and $y_{i}=0$ indicates no infectious complication. This optimization yielded the final weight vector:

$$w^{*}=(15,10,8,50,5)$$

Accordingly, the final RIRS-STAMP score was calculated as:

$${\mathrm{RIRS}\text{-}\mathrm{STAMP}}_{i}=15S_{i}+10T_{i}+8A_{i}+50M_{i}+5J_{i}$$

Thus, 15 points were assigned for stone size ≥20 mm, 10 points for operative time ≥60 min, 8 points for age ≥45 years, 50 points for recent preoperative UTI requiring antibacterial treatment, and 5 points for preoperative JJ stent placement. Estimated postoperative infectious risks were calculated from the logistic calibration model using the final RIRS-STAMP score as the predictor:

$$p_{i}=\frac{1}{1+e^{-(\beta 0+\beta 1\times score)}}$$

For presentation, model-based predicted probabilities were rounded into clinically interpretable risk intervals. Higher scores indicate an increased risk of postoperative infectious complications.

Abbreviations: STAMP, Stone size, operative Time, Age, Microbial history/recent preoperative UTI requiring antibacterial treatment, and Preoperative JJ stent; UTI, urinary tract infection.

For access: https://rirsstamp.pythonanywhere.com/ (accessed on 21 April 2026).

## 4. Discussion

The present study demonstrated an overall infectious complication rate of 8.1% in a large two-center cohort, which is consistent with previously reported rates of febrile urinary tract infection and urosepsis following RIRS [2,3,4,5]. Although RIRS is considered a safe and minimally invasive procedure, postoperative infection remains a major clinical concern [13]. This risk is likely driven by elevated intrarenal pressure during the procedure, facilitating pyelovenous and pyelotubular reflux and promoting bacterial translocation into the systemic circulation. Therefore, identifying both patient-related and procedure-related risk factors is essential for effective risk stratification and targeted preventive strategies [14].

While overall infection rates were similar between the two groups, a notable difference in infection phenotype was observed. Group 1 exhibited a higher rate of late-onset infections and readmissions, whereas Group 2 demonstrated a higher incidence of major infectious morbidity, including sepsis and septic shock. This pattern is partly consistent with prior experimental and clinical studies showing that manual or pressurized irrigation systems may generate higher and more variable intrarenal pressures than gravity-based systems, thereby potentially increasing the risk of bacteremia, systemic inflammatory response, and severe infectious events [6,11,12]. Importantly, the primary aim of this study was not causal comparison between the two centers or irrigation modalities, but development of a pooled risk score using the combined cohort; accordingly, the between-group findings should be interpreted as descriptive and hypothesis-generating However, our finding that total infection rates did not differ significantly between groups is not fully concordant with studies emphasizing irrigation-related pressure as a principal determinant of postoperative infectious morbidity [12]. This discrepancy suggests that irrigation modality alone may not determine whether infection occurs, but may instead influence its timing or severity, while center-specific practices and unmeasured confounders may also contribute.

Although late infections may partially reflect post-discharge factors and variability in follow-up practices, biological mechanisms such as stent-associated biofilm formation, prolonged stent dwell time, transient urinary stasis, residual stone fragments, and delayed bacterial release likely contribute to this pattern. These findings support a temporally dynamic and multifactorial model of post-RIRS infection, in which early and late infectious events may arise from distinct underlying mechanisms [15].

Operative duration emerged as a consistent predictor of infectious complications. Prolonged operative time has been associated with increased intrarenal pressure exposure and enhanced bacterial translocation, particularly in procedures exceeding 60 min [16,17]. Our findings are in line with previous reports and further support the importance of minimizing operative duration. In selected cases with high stone burden or procedural complexity, staged interventions may represent a safer approach to reduce infection risk [17,18].

Stone burden also plays a dual role in infection risk. Larger stones not only prolong operative time but may also serve as reservoirs for bacterial colonization and biofilm formation [2]. This microbial component is particularly relevant, as bacteria embedded within stone matrices or biofilms may evade detection in standard preoperative urine cultures, leading to underestimation of infection risk. Therefore, stone size should be interpreted not only as an anatomical parameter but also as a surrogate marker for microbial burden and procedural complexity [18].

Irrigation modality is another critical determinant of intrarenal pressure and infection risk [19]. Experimental and clinical data suggest that manual or pressurized irrigation systems generate higher and more variable pressure profiles compared to gravity-driven systems, potentially increasing the risk of bacteremia and systemic inflammatory response [20,21,22]. However, in the present study, irrigation modality was fully confounded with center, limiting causal interpretation. The observed differences in infection phenotype should therefore be considered hypothesis-generating. Future prospective studies incorporating direct intrarenal pressure measurements and standardized perioperative protocols are needed to clarify the independent contribution of irrigation strategy.

The role of sex as a predictor of infection was inconsistent across groups, suggesting potential effect modification or unmeasured confounding factors. Differences in comorbidity profiles, stent dwell time, perioperative antibiotic strategies, or institutional practices may explain this variability. These findings highlight the complexity of infection risk and the need for cautious interpretation of isolated predictors.

Preoperative JJ stenting was independently associated with postoperative infection, underscoring its clinical importance. Indwelling stents provide a favorable surface for bacterial adherence and biofilm formation, which may persist despite antibiotic prophylaxis [23]. Furthermore, microbial colonization within the upper urinary tract may not be adequately detected by midstream urine cultures. Previous studies have demonstrated that stone cultures may yield higher positivity rates compared to urine cultures, suggesting that conventional diagnostic approaches may underestimate microbial burden [24,25]. In this context, intraoperative renal pelvis or stone cultures may offer additional value in selected high-risk patients and support more tailored antimicrobial strategies [23,25].

In our study, *E. coli* was the predominant pathogen in both groups, while non-*E. coli* organisms such as *P. aeruginosa*, *E. faecalis*, and *K. pneumoniae* were also common. This pattern is consistent with previous reports in stone disease and endourological patients [26,27]. Clinically, this is relevant because a positive preoperative urine culture is a strong predictor of postoperative infectious complications; a meta-analysis of 5597 patients identified both positive urine culture and preoperative stenting as significant risk factors for urosepsis after ureteroscopy (OR 3.56 and 3.94, respectively) [28]. Group 2 also had a higher rate of polymicrobial cultures, although the significance of mixed flora remains unclear, as it may represent contamination, colonization, or true infection. Since recent data suggest that mixed-flora cultures alone may not increase postoperative infection risk [29], this finding should be interpreted cautiously.

A history of recent preoperative UTI treated with antibiotics was the strongest predictor of postoperative infection in our cohort. This variable should be interpreted as a marker of recent infection burden and underlying infectious susceptibility rather than as evidence that antibiotic treatment itself increases postoperative infection risk. Also this association likely reflects persistent microbial reservoirs or underlying host susceptibility rather than a direct causal effect of antibiotic exposure. Residual bacteria within stone matrices, biofilms, or resistant microbial populations may persist despite treatment, contributing to postoperative infection risk [2]. Given the potential for confounding by indication, these findings should be interpreted as indicators of increased risk rather than precise causal estimates.

Based on these observations, we developed the RIRS-STAMP score as a simplified perioperative risk-stratification tool derived from routinely available variables. Importantly, the modest discriminative performance was observed in the univariable ROC analyses of the individual continuous predictors (AUCs 0.55–0.58) and should not be conflated with the performance of the combined model. When these variables were integrated into the multivariable score, the full model showed good overall discrimination (AUC 0.84, 95% CI 0.80–0.88). Nevertheless, because the score was retrospectively derived and internally validated within the same dataset, it should be considered an adjunct to clinical judgment rather than a stand-alone decision-making tool, and prospective external validation in independent cohorts is required before routine clinical implementation.

Compared with previously published infection-focused models after RIRS, the discriminative performance of RIRS-STAMP appears encouraging, although direct comparison should be made cautiously because published tools differ in endpoints, predictor sets, and patient selection. For example, Yang et al. reported a nomogram for postoperative urosepsis in patients with negative preoperative urine culture with AUCs of 0.887 in the training cohort and 0.864 in the validation cohort; Senel et al. reported a febrile UTI score with an AUC of 0.837; and Hsieh et al. showed that the MAP score predicted postoperative fever and sepsis with AUCs of 0.798 and 0.799, respectively. In contrast, earlier attempts to repurpose general stone-complexity scores for infectious complications showed more limited performance, with the R.I.R.S. score achieving an AUC of 0.619 in one study. These data suggest that RIRS-STAMP has potentially competitive performance, but only external head-to-head validation can determine its incremental value over existing tools [17,30,31,32].

The strengths of this study include its large sample size, multicenter design, and the use of well-defined infectious outcomes. In addition, the development of a clinically applicable risk scoring system enhances its translational relevance. However, several limitations should be acknowledged. The retrospective design introduces the possibility of unmeasured confounding. The complete overlap between irrigation modality and study center precludes causal inference regarding irrigation effects. Furthermore, intrarenal pressure measurements, irrigation flow parameters, and intraoperative microbiological data (such as stone or renal pelvis cultures) were not available. Another important limitation of our study is that the proposed RIRS-STAMP risk score has not yet been externally validated in an independent cohort. Therefore, its generalizability and predictive performance across different patient populations should be confirmed by future prospective, multicenter external validation studies. Finally, certain relevant variables, including stent dwell time, could not be incorporated into the analysis.

Despite these limitations, the consistent identification of age, operative duration, preoperative stenting, and recent UTI as key predictors reinforces their importance in clinical practice. These factors should be prioritized when evaluating patients undergoing RIRS, particularly in the context of infection prevention and individualized perioperative management.

## 5. Conclusions

Our findings suggest that infectious complications after RIRS are multifactorial, arising from the interplay of patient susceptibility, procedural burden, and microbial factors. Stone size and operative duration were the variables most consistently associated with increased infection risk in this cohort. These findings support careful perioperative planning and closer monitoring, particularly in patients with pre-existing stents or a history of urinary tract infection. Although the observed association between irrigation method and infection severity is biologically plausible, potentially through increased intrarenal pressure and bacterial translocation, causality cannot be established from the present study and should be evaluated in prospective studies. Finally, the RIRS-STAMP score should be considered a preliminary tool for early risk stratification and individualized perioperative management, particularly for infection prevention and postoperative monitoring; however, it requires prospective validation before routine clinical use.

## Figures and Tables

**Table 1:** Demographic and clinical characteristics.

Parameter	Total (n = 1949)	Group 1 (n = 1112)	Group 2 (n = 837)	Effect Estimate (95% CI)	*p* Value
Sex, n (%)				OR: 2.99 (95% CI, 2.48 to 3.60)	^a^ <0.001
Male	1021 (52.4)	710 (63.8)	311 (37.2)		
Female	928 (47.6)	402 (36.2)	526 (62.8)		
Age, years	41.52 ± 17.14; 43 (33–54)	39.09 ± 18.31; 41 (32–52)	44.73 ± 14.88; 45 (35–55)	−5.64 years (95% CI, −7.12 to −4.16)	^b^ <0.001
Laterality, n (%)				OR: 1.02 (95% CI, 0.85 to 1.22)	^a^ 0.873
Left RIRS	1018 (52.2)	583 (52.4)	435 (52.0)		
Right RIRS	931 (47.8)	529 (47.6)	402 (48.0)		
Stone size, mm, median (IQR)	15 (11–23)	15 (10–24)	17 (12–23)	(95% CI, −3 to −1)	^c^ 0.016 *
Stone size category, n (%)				OR: 1.03 (95% CI, 0.86 to 1.24)	^a^ 0.790
<20 mm	1116 (57.3)	633 (56.9)	483 (57.7)		
≥20 mm	833 (42.7)	479 (43.1)	354 (42.3)		
Stone location, n (%)				Cramér’s V: 0.23 (95% CI, 0.18 to 0.27)	^a^ <0.001
Pelvis	648 (33.2)	413 (37.1)	235 (28.1)		
Lower calyx	524 (26.9)	292 (26.3)	232 (27.7)		
Middle calyx	125 (6.4)	63 (5.7)	62 (7.4)		
Upper calyx	87 (4.5)	47 (4.2)	40 (4.8)		
Ureter	110 (5.6)	16 (1.4)	94 (11.2)		
Multiple	455 (23.4)	281 (25.3)	174 (20.8)		
Access sheath, n (%)				OR: 0.91 (95% CI, 0.60 to 1.40)	^a^ 0.588
No	93 (4.8)	55 (4.9)	38 (4.5)		
Yes	1856 (95.2)	1057 (95.1)	799 (95.5)		
Preoperative JJ stent, n (%)				OR: 4.90 (95% CI, 3.99 to 6.02)	^a^ <0.001
No	622 (31.9)	195 (17.5)	427 (51.0)		
Yes	1327 (68.1)	917 (82.5)	410 (49.0)		
Postoperative JJ stent, n (%)				OR: 0.79 (95% CI, 0.58 to 1.08)	^a^ 0.116
No	190 (9.7)	118 (10.6)	72 (8.6)		
Yes	1759 (90.3)	994 (89.4)	765 (91.4)		
Stone-free status, n (%)				OR: 1.76 (95% CI, 1.31 to 2.36)	^a^ <0.001
Yes	1749 (89.7)	1023 (92.0)	726 (86.7)		
No	200 (10.3)	89 (8.0)	111 (13.3)		
Operative time, min, median (IQR)	50 (35–65)	40 (25–60)	60 (45–70)	(95% CI, −19.5 to −15)	^c^ <0.001
Operative time category, n (%)				OR: 2.94 (95% CI, 2.43 to 3.55)	^a^ <0.001
<60 min	1198 (61.5)	804 (72.3)	394 (47.1)		
≥60 min	751 (38.5)	308 (27.7)	443 (52.9)		
Length of hospital stay, day	1.36 ± 1.26	1.37 ± 1.08	1.35 ± 1.47	(95% CI, −0.10 to 0.14)	^b^ 0.326
Preoperative urine culture, n (%)				OR: 1.32 (95% CI, 0.82 to 2.14)	^a^ 0.252
No	1875 (96.2)	1065 (95.8)	810 (96.8)		
Yes	74 (3.8)	47 (4.2)	27 (3.2)		
Antibacterial treatment of preoperative UTI, n (%)				OR: 3.77 (95% CI, 2.44 to 5.82)	^a^ <0.001
No	1803 (92.5)	992 (89.2)	811 (96.9)		
Yes	146 (7.5)	120 (10.8)	26 (3.1)		
Infectious complications, n (%)	158 (8.1)	95 (8.54)	63 (7.53)	OR: 1.15 (95% CI, 0.82 to 1.60)	^d^ 0.451

^a^ Chi-square analysis, ^b^ Independent samples *t*-test. * *p* < 0.05 ^c^ Mann-Whitney U Test. ^d^ Two-sided Fisher Exact test. Abbreviations: CI, confidence interval; IQR, interquartile range; OR, odds ratio; RIRS, retrograde intrarenal surgery; UTI, urinary tract infection.

**Table 2:** Comparison of infectious outcomes between groups.

	Group 1 (n = 1112) n (%)	Group 2 (n = 837) n (%)	OR (95% CI)	^ *p*
Sepsis	10 (0.90)	15 (1.79)	0.50 (0.22–1.11)	0.103
Septic shock	3 (0.27)	8 (0.96)	0.28 (0.07–1.06)	0.064
Early UTI (≤48 h)	22 (1.98)	12 (1.43)	1.39 (0.68–2.82)	0.388
Late UTI (>48 h)	60 (5.40)	28 (3.35)	1.65 (1.04–2.60)	0.036 *
Major UTI (Sepsis + Septic shock)	13 (1.17)	23 (2.75)	0.42 (0.21–0.83)	0.016 *
Total UTI	95 (8.54)	63 (7.53)	1.15 (0.82–1.60)	0.451

^^^ Two-sided Fisher Exact test. * *p* < 0.05. Abbreviation: UTI: urinary Tract Infection.

**Table 3:** Results of univariate and multivariate logistic regression analysis of factors affecting the development of infection.

Groups	Parameters	Factors Affecting the Development of UTI			
		Univariate Analysis		Multivariate Analysis	
		OR (95% CI)	*p* value	OR (95% CI)	*p* value
Group 1 + Group 2 (n = 1949)	Age (year)	1.01 (1.01–1.02)	0.003 *	1.01 (1.00–1.02)	0.045 *
	Stone Size (mm)	1.02 (1.01–1.04)	0.013 *	1.01 (0.99–1.03)	0.154
	Preoperative JJ Stent	1.37 (0.95–1.97)	0.014 *	1.48 (1.02–2.15)	0.038 *
	Operation Time (min)	1.01 (1.00–1.02)	0.041 *	1.01 (1.00–1.02)	0.049 *
	Antibacterial treatment of preoperative UTI	75.98 (49.6–116.3)	<0.001 *	68.45 (44.2–105.8)	<0.001 *

Result of Logistic Regression Analysis * *p* < 0.05. Model Summary R^2^: 0.564. Abbreviations: OR: Odds ratio; UTI: Urinary Tract Infection.

**Table 4:** ROC analysis of variables including age, stone size, and operation time in predicting the development of infection.

Groups	Parameters	Cut-Off Values	Sensitivity (%)	Specificity (%)	AUC (95% CI)	*p* Value
Group 1 + Group 2	Stone size (mm)	19.5	49.4	57.8	0.56 (0.52–0.61)	0.013 *
	Age (years)	43.5	55.3	55.2	0.58 (0.53–0.62)	0.003 *
	Operation Time (min)	59.5	44.9	62	0.55 (0.50–0.59)	0.041 *

* *p* < 0.05.

**Table 5:** The RIRS-STAMP scoring system for predicting postoperative infectious complications: A point-based risk assessment model.

Variables	Status	Point
Stone Size (mm)	≥20	15
Operation time (min)	≥60	10
Age (years)	≥45	8
Antibacterial treatment of preoperative UTI	Yes	50
Preoperative JJ Stent	Present	5

**Table 6:** Observed and estimated postoperative infectious risk according to RIRS-STAMP score category.

Risk Categories	Patients, n (%)	Observed Major UTI, n/N (%)	Observed Total UTI, n/N (%)	Estimated Major UTI Risk (%)	Estimated Total UTI Risk (%)
Low risk (0–15 points)	1033 (53.0)	4/1033 (0.39)	27/1033 (2.6)	<0.5	<3
Moderate risk (16–40 points)	770 (39.5)	12/770 (1.6)	54/770 (7)	1.0–1.5	5–15
High risk (41–65 points)	75 (3.9)	5/75 (6.6)	31/75 (41.3)	2.5–5.0	20–40
Very high risk (>65 points)	71 (3.6)	15/71 (21.1)	46/71 (64.7)	>10	>50

**Table 7:** Clinical management recommendations based on RIRS-STAMP risk stratification.

Risk Category	Clinical Recommendation
Low risk	Standard prophylaxis may be sufficient; routine follow-up.
Moderate risk	Closer observation may be considered, particularly for delayed infectious events.
High risk	Elevated risk; intensified inpatient monitoring may be considered.
Very high risk	Early escalation and individualized preventive strategies should be considered, including ICU preparedness.

## Data Availability

The data that support the findings of this study are available from the corresponding author upon reasonable request.

## Abbreviations

The following abbreviations are used in this manuscript:

AUC	Area under the curve
CIRF	Clinically insignificant residual fragments
ICU	Non-contrast computed tomography
NCCT	Odds ratio
OR	Retrograde intrarenal surgery
RIRS	Intensive care unit
SPSS	Statistical Package for the Social Sciences
UAS	Ureteral access sheath
UTI	Urinary tract infection
VIF	Variance inflation factor

## Appendix A

**Table A1:** Distribution of isolated microorganisms according to study groups.

Microorganism	Group 1 (n = 47) n (%)	Group 2 (n = 27) n (%)	Total (n = 74) n (%)	*p* Value
*Escherichia coli*	21 (42.9%)	12 (34.3%)	33 (39.3%)	—
*Klebsiella pneumoniae*	9 (18.4%)	4 (11.4%)	13 (15.5%)	—
*Pseudomonas aeruginosa*	5 (10.2%)	7 (20.0%)	12 (14.3%)	—
*Enterococcus faecalis*	4 (8.2%)	6 (17.1%)	10 (11.9%)	—
*Proteus* spp.	3 (6.1%)	1 (2.9%)	4 (4.8%)	—
*Candida* spp.	3 (6.1%)	0 (0.0%)	3 (3.6%)	—
*Enterococcus faecium*	1 (2.0%)	1 (2.9%)	2 (2.4%)	—
*Staphylococcus epidermidis*	1 (2.0%)	1 (2.9%)	2 (2.4%)	—
*Acinetobacter baumannii*	0 (0.0%)	1 (2.9%)	1 (1.2%)	—
*Corynebacterium* spp.	1 (2.0%)	0 (0.0%)	1 (1.2%)	—
*Enterobacter cloacae*	1 (2.0%)	0 (0.0%)	1 (1.2%)	—
*Serratia marcescens*	0 (0.0%)	1 (2.9%)	1 (1.2%)	—
*Staphylococcus aureus*	0 (0.0%)	1 (2.9%)	1 (1.2%)	—
Polymicrobial cultures	2 (4.3%)	7 (25.9%)	9 (12.2%)	0.01 *

Note: The comparison of polymicrobial cultures was performed on a sample basis using Fisher’s exact test (* *p* < 0.05). Species-level *p* values were not calculated because the microorganism distribution was isolate-based and several cells had sparse counts.

**Table A2:** Clinical and demographic parameters associated with the development of infection.

	Group 1 (n = 1112)			Group 2 (n = 837)			Group 1 + Group 2 (n = 1949)		
Parameters	Infection (−) (n = 1017)	Infection (+) (n = 95)	*p* Value	Infection (−) (n = 774)	Infection (+) (n = 63)	*p* Value	Infection (−) (n = 1791)	Infection (+) (n = 158)	*p* Value
Sex n (%)									
Male	668 (65.6)	42 (44.2)	^a^ 0.001 *	275 (35.5)	36 (57.2)	^a^ 0.001 *	943 (52.7)	78 (49.4)	^a^ 0.477
Female	349 (34.4)	53 (55.8)		499 (64.5)	27 (42.8)		848 (47.3)	80 (50.6)	
Age, years (mean ± SD)	38.72 ± 18.26	44.25 ± 18.37	^b^ 0.006 *	44.58 ± 14.92	46.60 ± 14.31	^b^ 0.148	41.23 ± 17.15	45.33 ± 16.61	^b^ 0.003 *
Laterality (n, %)									
Left RIRS	533 (52.4)	50 (52.6)	^a^ 0.952	402 (51.9)	33 (52.4)	^a^ 0.848	935 (52.2)	83 (52.5)	^a^ 0.934
Right RIRS	484 (47.6)	45 (47.4)		372 (48.1)	30 (47.6)		856 (47.8)	75 (47.5)	
Stone size (mm) (mean ± SD)	17.88 ± 9.02	21.14 ± 9.38	^b^ 0.001 *	18.60 ± 8.20	18.45 ± 9.56	^b^ 0.902	18.19 ± 8.68	19.90 ± 9.52	^b^ 0.013 *
Stone size (n, %)									
<20 mm	589 (57.9)	44 (46.3)	^a^ 0.049 *	447 (57.8)	36 (57.1)	^c^ 0.986	1036 (57.8)	80 (50.6)	^a^ 0.09
≥20 mm	428 (42.1)	51 (53.7)		327 (42.2)	27 (42.9)		755 (42.2)	78 (49.4)	
Stone location (n, %)									
Pelvis	380 (37.3)	33 (34.7)	^a^ 0.832	216 (27.9)	19 (30.2)	^a^ 0.805	596 (33.3)	52 (32.9)	^a^ 0.878
Lower calyx	267 (26.3)	25 (26.3)		215 (27.8)	17 (27)		482 (26.9)	42 (26.6)	
Middle calyx	59 (5.8)	4 (4.2)		59 (7.6)	3 (4.8)		118 (6.6)	7 (4.4)	
Upper calyx	45 (4.4)	2 (2.1)		35 (4.5)	5 (7.9)		80 (4.4)	7 (4.4)	
Ureter	15 (1.5)	1 (1.1)		88 (11.4)	6 (9.5)		103 (5.8)	7 (4.4)	
Multiple	251 (24.7)	30 (32.6)		161 (20.8)	13 (20.6)		412 (23)	43 (27.3)	
Access Sheath									
No	52 (5.1)	3 (3.2)	^c^ 0.346	36 (4.7)	2 (3.2)	^c^ 0.600	88 (4.9)	5 (3.2)	^c^ 0.288
Yes	965 (94.9)	92 (96.8)		738 (95.3)	61 (96.8)		1703 (95.1)	153 (96.8)	
Preoperative JJ Stent (n, %)									
No	183 (18.0)	12 (12.6)	^a^ 0.185	398 (51.4)	29 (46)	^a^ 0.468	581 (32.4)	41 (25.9)	^a^ 0.014 *
Yes	834 (82.0)	83 (87.4)		376 (48.6)	34 (54)		1210 (67.6)	117 (74.1)	
Postoperative JJ Stent (n, %)									
No	110 (10.8)	8 (8.4)	^c^ 0.447	66 (8.5)	6 (9.5)	^c^ 0.790	176 (9.8)	14 (8.9)	^a^ 0.660
Yes	907 (89.2)	87 (91.6)		708 (91.6)	57 (90.5)		1615 (90.2)	144 (91.1)	
Stone free (n, %)									
Yes	938 (92.2)	85 (89.5)	^a^ 0.379	674 (87.1)	52 (82.5)	^c^ 0.319	1612 (90)	137 (86.7)	^a^ 0.162
No	79 (7.8)	10 (10.5)		100 (12.9)	11 (17.5)		179 (10)	21 (13.3)	
Operative time (min) (mean ± SD)	42.90 ± 19.34	46.97 ± 19.17	^b^ 0.039 *	58.35 ± 17.62	58.67 ± 18.45	^b^ 0.446	49.50 ± 20.13	52.35 ± 19.67	^b^ 0.041 *
Operative time (n, %)									
<60 min	746 (73.4)	58 (61.1)	^a^ 0.024 *	365 (47.2)	29 (46)	^a^ 0.958	1111 (62)	87 (55.1)	^a^ 0.078
≥60 min	271 (26.6)	37 (38.9)		409 (52.8)	34 (54)		680 (38)	71 (44.9)	
Length of hospital stay (day) (mean ± SD)	1.21 ± 0.68	3.65 ± 2.33	^b^ 0.001 *	1.07 ± 0.48	4.73 ± 3.66	^b^ 0.001 *	1.15 ± 0.61	4.15 ± 3.06	^b^ 0.001 *
Antibacterial treatment of preoperative UTI									
No	974 (95.8)	18 (18.9)	^c^ 0.001 *	774 (100)	37 (57.7)	^c^ 0.001 *	1748 (97.6)	55 (34.8)	^a^ 0.001 *
Yes	43 (4.2)	77 (81.1)		0 (0)	26 (42.3)		43 (2.4)	103 (65.2)	
									

^a^ Chi-square analysis, ^b^ Independent samples *t*-test, ^c^ Fisher’s Exact Test. * *p* < 0.05. Abbreviations: UTI: urinary tract infection.

**Table A3:** Class-specific performance metrics of the final score.

Risk Categories	Sensitivity	Specificity	PPV	NPV
Low risk (0–15 points)	22.1%	91.5%	2.8%	97.2%
Moderate risk (16–40 points)	45.6%	84.2%	10.4%	94.1%
High risk (41–65 points)	68.4%	72.8%	24.8%	95.3%
Very high risk (>65 points)	65.2%	97.6%	70.5%	96.8%
